# Reply to Comment on ‘Statin use and all-cancer survival: prospective results from the Women’s Health Initiative’

**DOI:** 10.1038/bjc.2016.396

**Published:** 2016-12-06

**Authors:** Ange Wang, Aaron K Aragaki, Jean Y Tang, Allison W Kurian, JoAnn E Manson, Rowan T Chlebowski, Michael Simon, Pinkal Desai, Sylvia Wassertheil-Smoller, Simin Liu, Stephen Kritchevsky, Heather A Wakelee, Marcia L Stefanick

**Affiliations:** 1 Department of Medicine, Division of Oncology, Stanford University School of Medicine, Stanford, CA 94305, USA; 2 Division of Public Health Sciences, Fred Hutchinson Cancer Research Center, Seattle, WA 98109, USA; 3 Department of Dermatology, Stanford University School of Medicine, Stanford, CA 94305, USA; 4 Department of Health Research and Policy, Stanford University School of Medicine, Stanford, CA 94305, USA; 5 Department of Medicine, Brigham and Women’s Hospital, Harvard Medical School, Boston, MA 02115, USA; 6 Department of Hematology/Oncology, Los Angeles Biomedical Research Institute at Harbor-UCLA Medical Center, Torrance, CA 90502, USA; 7 Department of Oncology, Karmanos Cancer Institute at Wayne State University, Detroit, MI 48201, USA; 8 Department of Medicine, Weill Cornell Medical College, New York, NY 10065, USA; 9 Department of Epidemiology and Population Health, Albert Einstein College of Medicine, Bronx, NY 10461, USA; 10 Department of Medicine, Brown University School of Medicine, Providence, RI 02912, USA; 11 Department of Internal Medicine, Wake Forest School of Medicine, Winston-Salem, NC 27101, USA; 12 Department of Medicine, Stanford Prevention Research Center, Stanford University School of Medicine, Stanford, CA 94035, USA

**Sir,**

In a cohort of 146 326 postmenopausal women in the Women’s Health Initative, our study (Wang *et al*, 2016) found that regular use of statins or other lipid-lowering medications was associated with decreased cancer death. Many studies have suggested that statin use may be associated with lower risk of cancer incidence and increased survival in multiple cancer types. A meta-analysis of 990 649 patients found that statin use after diagnosis was associated with significantly decreased all-cancer mortality (Zhong *et al*, 2015). Another meta-analysis of 523 193 patients reported that statin use was associated with significantly reduced all-cause mortality in cancer patients (HR 0.82, 9% CI 0.76–0.89; Li *et al*, 2015). Our study reported similar findings as a large retrospective Danish study of 295 925 cancer patients, which reported that statin users had 15% reduction in all-cancer mortality (HR 0.85, 95% CI 0.82–0.87; Nielsen *et al*, 2012). Multiple studies have also reported a possible protective effect for statins on specific cancer types (Friis *et al*, 2005; Fortuny *et al*, 2006; Farwell *et al*, 2008; Nowakowski *et al*, 2010; Simon *et al*, 2012; Singh and Singh, 2013a, 2013b; Wu *et al*, 2013; Singh *et al*, 2013a, 2013b; Gaist *et al*, 2013, 2014; Ling *et al*, 2015; Nevadunsky *et al*, 2015). However, though many studies have suggested that statins may decrease cancer incidence and mortality, not all studies have found this effect (Dale *et al*, 2006; Cholesterol Treatment Trialists C, 2015), including the studies cited in the Comment.

In response to the comment that our article might have selected the healthy statin user or unselected the unhealthy cancer patients with low cholesterol, our article extensively controlled for potential confounders including age, race/ethnicity, education, smoking, body mass index, physical activity, family history of cancer, current health care provider, oral contraception use, prior unopposed oestrogen use, prior oestrogen plus progestin use, solar irradiance (latitude), prior CHD history, prior diabetes history, randomisation into the CaD trial and age at menarche. Although healthy bias cannot be entirely excluded, we believe our analysis was as robust as possible in extensively controlling for confounders. The Women’s Health Initative is a large and well-validated data set. In addition, our findings are similar as the large Dutch study which gives us additional confidence in our results. However, we have been careful to state that our article can only establish associations and not causal links between statins and cancer.

Multiple biological mechanisms have been proposed for a possible protective effect of statins on cancer, including the following: blocking the mevalonate pathway which may interfere with cell proliferation and migration; (Fenton *et al*, 1992; Herold *et al*, 1995; Deberardinis *et al*, 2008; Boudreau *et al*, 2010) disruption of G-protein expression (Wong *et al*, 2002; Demierre *et al*, 2005), pro-apoptotic properties through regulation of the RAF-mitogen-activated protein kinase 1 pathway (Wu *et al*, 2004); and arresting the cell cycle (Crick *et al*, 1998). However, as our article states, these mechanisms warrant further investigation on which one(s) are the critical drivers of the relationship between statins and cancer.

Overall given the conflicting evidence in literature, we reiterate that the link between statins and cancer incidence and mortality should be further investigated in randomized controlled trials (RCTs). The literature has reported protective effects of statins and cancer in many but not all studies, and our article contributes to the extensive literature on this topic. We recognise that not all articles have found a protective effect, and that this topic warrants extensive further investigation. RCTs with cancer outcomes as the primary outcome are particularly important in further elucidating this relationship.
